# Bystander effects in UV-induced genomic instability: Antioxidants inhibit delayed mutagenesis induced by ultraviolet A and B radiation

**DOI:** 10.1186/1477-3163-4-11

**Published:** 2005-08-09

**Authors:** Jostein Dahle, Egil Kvam, Trond Stokke

**Affiliations:** 1 Department of Radiation Biology, The Norwegian Radium Hospital, Montebello, 0310 OSLO, Norway

## Abstract

**Background:**

Genomic instability is characteristic of many types of human cancer. Recently, we reported that ultraviolet radiation induced elevated mutation rates and chromosomal instability for many cell generations after ultraviolet irradiation. The increased mutation rates of unstable cells may allow them to accumulate aberrations that subsequently lead to cancer. Ultraviolet A radiation, which primarily acts by oxidative stress, and ultraviolet B radiation, which initially acts by absorption in DNA and direct damage to DNA, both produced genomically unstable cell clones. In this study, we have determined the effect of antioxidants on induction of delayed mutations by ultraviolet radiation. Delayed mutations are indicative of genomic instability.

**Methods:**

Delayed mutations in the *hypoxanthine phosphoribosyl transferase *(*hprt*) gene were detected by incubating the cells in medium selectively killing *hprt *mutants for 8 days after irradiation, followed by a 5 day period in normal medium before determining mutation frequencies.

**Results:**

The UVB-induced delayed *hprt *mutations were strongly inhibited by the antioxidants catalase, reduced glutathione and superoxide dismutase, while only reduced glutathione had a significant effect on UVA-induced delayed mutations. Treatment with antioxidants had only minor effects on early mutation frequenies, except that reduced glutathione decreased the UVB-induced early mutation frequency by 24 %. Incubation with reduced glutathione was shown to significantly increase the intracellular amount of reduced glutathione.

**Conclusion:**

The strong effects of these antioxidants indicate that genomic instability, which is induced by the fundamentally different ultraviolet A and ultraviolet B radiation, is mediated by reactive oxygen species, including hydrogen peroxide and downstream products. However, cells take up neither catalase nor SOD, while incubation with glutathione resulted in increased intracellular levels of glutathione. Previously, we have shown that ultraviolet induced delayed mutations may be induced via a bystander effect and that this effect is 5-fold higher for UVB radiation than for UVA radiation. Therefore, we propose that the antioxidants inhibit an ultraviolet radiation-induced bystander effect and that the effect is transmitted via the medium and via an internal transfer between cells, like gap junctional intercellular communication, for UVB radiation and only by the latter mechanism for UVA radiation.

## Background

Genomic instability is a hallmark of multistage carcinogenesis [1]. The instability is likely to accelerate the mutation rate of unstable cells and give them growth advantage over normal cells. It is established that ionizing radiation causes genomic instability in mammalian cells in the form of a persistent increase in various types of genetic changes such as mutations, sister chromatid exchanges and chromosome aberrations that last for many cell generations after exposure [2]. The mechanisms of induction and maintenance of these persistent changes are largely unknown. However, it is likely that ionizing radiation-induced genomic instability is maintained for many cell generations by factors such as a persistent induction of DNA damage by increased levels of oxidative stress, mutations in DNA repair enzymes or radiation-induced up regulation of error-prone DNA repair mechanisms [3].

Ionizing radiation causes only a minor fraction of human cancers, and the types and locations of cellular damage by ionizing radiation are different from those of other environmental stress factors or endogenous stress [4,5]. We have chosen to use induction of genomic instability by ultraviolet A (UVA radiation, 320 – 400 nm) and ultraviolet B radiation (UVB radiation, 290–320 nm) as our model system [6]. UVB radiation is an established complete carcinogen that is the main cause of non-melanoma skin cancers such as squamous cell carcinomas [7]. UVA radiation is suspected to play a role in induction of genomically unstable malignant melanoma [8,9]. Both UVA and UVB radiation induce genomic instability in mammalian cells in the form of delayed gene mutations that occur 10–20 cell generations after exposure [6]. The unstable cell clones with delayed mutations were also chromosomally unstable.

UVB radiation acts initially by absorption in DNA and production of direct DNA damage in the form of pyrimidine dimers and other photoproducts [10]. In contrast, UVA radiation acts primarily in the presence of oxygen and endogenous photosensitizers by generating reactive oxygen species (ROS) in exposed cells [11]. Thus, UVA radiation induces a type of oxidative stress that resembles endogenous oxidative stress by inflammation or by other endogenous sources [12]. However, UVB radiation subsequently also induces oxidative stress to some degree, especially in the form of hydrogen peroxide and lipid peroxides [13,14], and UVA radiation also induce pyrimidine dimers [15]. Nevertheless, the spectra and time courses of UVA-induced ROS and DNA damage are very different from those of UVB [16].

ROS are involved in many aspects of biology, such as atherosclerosis, aging, mutagenesis and carcinogenesis [17]. Increased levels of ROS have been found in genomically unstable cell clones induced by ionizing radiation [18], and the antioxidant catalase has been shown to inhibit UVA-induced delayed formation of micronuclei [19]. Antioxidants reduce oxidation of biomolecules by ROS and other oxidants and may be a factor in reduction of the incidence of many types of human cancer, including skin cancer, by high consumption of tea, fruits and vegetables [20-22].

Reduced glutathione (GSH), catalase, and superoxide dismutase (SOD) are water-soluble antioxidants [23]. GSH reduces a broad spectrum of oxidants, whereas catalase and SOD specifically reacts with H_2_O_2 _and superoxide, respectively [23]. Catalase and SOD are not taken up by cells, but extracellular GSH can be broken down by the plasma membrane enzyme γ-glutamyltransferase (GGT), which removes the γ-glutamyl moiety, and the resulting products can be taken up by cells and stimulate intracellular GSH synthesis [31]. H_2_O_2 _is a weak oxidant and poorly reactive. However, it can be converted to the highly reactive hydroxyl radical via the iron catalysed Fenton reaction. H_2_O_2_might be produced by a number of different non-enzymatic or enzymatic (SOD, oxidases, peroxidases and hydrogenases) processes [23]. UV radiation can activate flavin-containing NAD(P)H oxidases which subsequently lead to production of H_2_O_2 _[24].

Recently it was shown that UV-induced delayed mutations might be induced via a bystander effect [25]. The bystander effect was 5-fold higher for UVB radiation than for UVA radiation. The factors mediating the bystander effect are unknown but reactive oxygen species are likely candidates. We hypothesize that antioxidants added after UVA and UVB radiation are potent inhibitors of genomic instability manifested as delayed mutations.

## Materials and methods

### Cell line and culture conditions

Chinese hamster fibroblasts (V79 cells) were cultured in RPMI 1640 medium (PAA, Austria) supplemented with 10 % FCS (PAA, Austria), penicillin (100 U/ml), streptomycin (100 μg/ml), L-glutamine (2 mM) and the following antioxidants (Sigma, Saint Louis, MO, USA): 1000 IU/ml catalase from a 20.000 IU/ml stock solution in PBS, 5 mM GSH from a stock solution of 0.1 M in PBS (pH adjusted to 7.4) or 500 IU/ml SOD from a stock solution of 7500 IU/ml in PBS.

### UV- measurements

**Figure 1:**
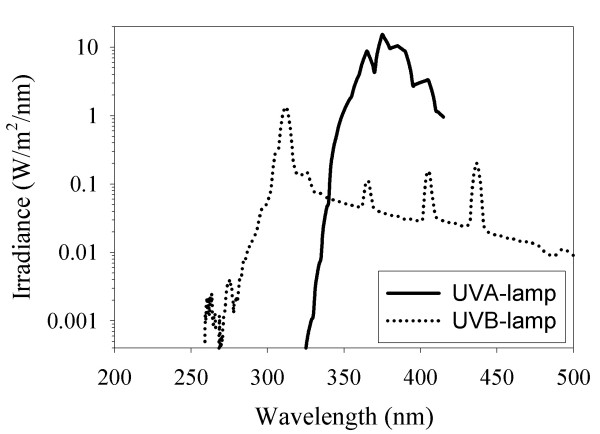
**Lamp spectra**. A) The UVA lamp has a peak at 375 nm. Total UVA- irradiance (320–400 nm) was 436 W/m^2^. B) The UVB lamp has a peak at 312 nm. Total UVB-irradiance (290–320 nm) was 23.3 W/m^2^.

The irradiance from the lamps was routinely measured with a PMA 2200 photometer (Solar light Co, Philadelphia, PA, USA) before each treatment. The lamp spectra (Figure 1) and absolute irradiance from the lamps were measured by an irradiance-calibrated USB 2000 spectrometer (Avantes, Eerbeek, Holland) as described previously [6].

### UV-irradiation protocol

Cells were seeded in 25 cm^2 ^flasks (Nalge Nunc, Naperville, IL, USA) the day before irradiation. The cells were irradiated from above in PBS. The UVA lamp contains a 3 kW Hg-Xe light bulb and filters to remove unwanted UVB radiation and visible light (Sellamed 3000 lamp, Sellamed, Gevelsberg, Germany). The irradiance from the UVA lamp was 436 W/m^2^. The flasks transmitted 392 W/m^2^. The UVB lamp contains a bank of six fluorescing tubes (TL-20W/01RS, Philips, Amsterdam, Holland). The irradiance from the UVB lamp was 23.3 W/m^2^. The flasks transmitted 18.6 W/m^2^.

### Measurement of survival

Eight hundred cells were seeded 18 h before exposure with UVA or UVB radiation. Antioxidants were added immediately after irradiation. The cells were incubated for 6–7 days after irradiation in cell medium, washed with 9 mg/ml NaCl, fixed with absolute alcohol and stained with a saturated methylene blue solution. Colonies larger than 50 cells were counted manually.

### Measurement of Early HPRT Mutations

Cells were irradiated with UVA or UVB radiation. Antioxidants were added immediately after irradiation. The cells were subcultured on the third and sixth day after irradiation. On the eight day after irradiation one million cells were seeded in 4–6 100 mm dishes in medium with 5 μg/ml 6-thioguanine (6TG) (Sigma, Saint Louis, MO, USA) and 200 cells in triplicate 60 mm dishes in medium without 6TG. Seven days after seeding the cells were washed, fixed and stained. Images were obtained of the dishes with a flatbed scanner and homemade software was used to count the colonies [26]. The mutation frequency, m, (number of mutants per million cells) was calculated by equation 1:



where M is the total number of mutants counted, C is the total number of cells seeded (usually 1 million) and c.e. is the cloning efficiency.

### Measurement of Delayed HPRT Mutations

The procedure used to determine delayed mutations has been described before [25]. Briefly, however, Cells were irradiated with UVA radiation or UVB radiation. Immediately after irradiation the PBS was replaced by medium containing HAT (0.2 mM hypoxanthine, 0.4 μM aminopterin and 75 μM thymidine) (Sigma, Saint Louis, MO, USA). Aminopterin (Sigma, Saint Louis, MO, USA) blocks de novo synthesis of DNA precursors, killing cells that lack the purine salvage pathway (HPRT mutants) whereas the wild type cells survive [27]. Four days after irradiation the cells were subcultured and again seeded in medium with HAT. Four days later the cells were subcultured again, but now incubated in ordinary medium (without HAT). After five days in ordinary medium, one million cells were seeded in 3–6 100 mm dishes per dose in medium with 5 μg/ml 6TG and 200 cells in triplicate 60 mm dishes per dose in medium without 6TG. Antioxidants were added immediately after irradiation and used until seeding in medium with 6TG. Six days after seeding the cells were washed, fixed, stained and counted as described above. The mutation frequency was calculated by equation 1. It may be conceivable that the delayed HPRT mutants detected were early mutations that were resistant to aminopterin treatment. However, when 13 different UV-and X radiation-induced HPRT- clones were tested for growth in HAT medium, only four cells in 25 million cells plated formed colonies after 7 days of growth. Thus, the assay used was valid for detecting delayed mutations.

### Flow cytometry

Cells were washed two times with PBS and incubated with 50 μM monochlorobimane (MCB) for 45 minutes as described previously [28]. Subsequently the cells were trypsinsed and stained with 1 μg/ml propidium iodide (PI). The samples were analyzed in a FACS^DIVA ^flow cytometer (Becton Dickinson, USA) equipped with one argon (Spectra Physics, USA) and one krypton laser (Coherent, USA) tuned to 488 nm and UV, respectively. Forward scatter (FSC), side scatter (SSC) and PI fluorescence (>630 nm) were measured at the first focus (488 nm, 200 mW). The acquisition was triggered on the FSC signal, and the area of the PI signal was measured and used to gate away dead cells. MCB fluorescence (465–505 nm) was excited with 50 mW of UV light (351/356 nm) at the second laser intercept.

### Statistics

Kruskal-Wallis one-way analysis of variance on ranks was used to test for differences among the treatment groups. Mann Whitney rank sum test, Dunn's method and student t-test were used for comparison of treated groups versus control groups. A significance level of 0.05 was used unless otherwise noticed.

## Results

To investigate the effect of reactive oxygen species produced after UVA or UVB radiation on early and delayed *hprt *mutations as well as cell survival, we treated irradiated cells with three different antioxidants. The cells were cultured in medium with antioxidants from immediately after irradiation and until seeding in selective medium for determination of mutant frequencies and until fixation for determination of cell survival.

### Survival of cells after UV radiation

High mutation frequencies were advantageous in order to get high sensitivity of the antioxidant treatment. Therefore, doses of 321 kJ/m^2^UVA-radiation and 8.1 kJ/m^2 ^UVB-radiation were chosen in the present study. This choice is based on figure 2 of ref [25], which shows that the fraction of early *hprt *mutants increases with the dose up to 321 kJ/m^2 ^for UVA radiation and up to 8.1 kJ/m^2 ^for UVB radiation, above which the mutant fraction reaches a plateau. For delayed mutations, 321 kJ/m^2 ^of UVA radiation and 11.3 kJ/m^2 ^of UVB radiation results in maximum delayed mutant frequency (Figure 2 of ref [25]). These doses resulted in around 36 % cell survival for UVA radiation and 33 % cell survival for UVB radiation (Table 1). The doses were physiologically relevant as they correspond to midday sun exposure times in midsummer of 2–5 hours in Finland and half an hour in Italy [29,30]

**Figure 2:**
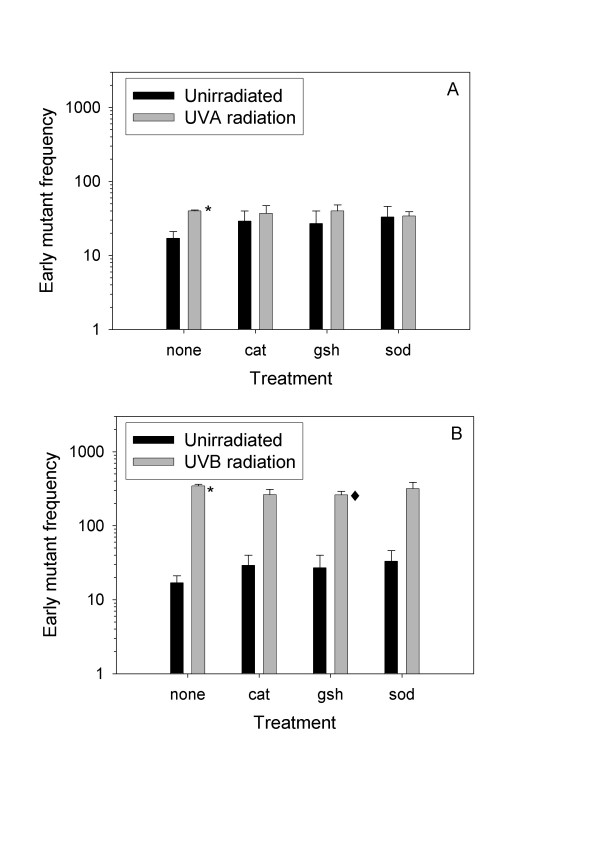
**Effect of antioxidants on UVA- and UVB-induced early mutagenesis**. Cells were exposed to 321 kJ/m^2 ^UVA- (A) or 8.1 kJ/m^2 ^UVB radiation (B). The cells were grown with or without antioxidants in ordinary medium for 8 days after ultraviolet irradiation. Subsequently, the cells were seeded in medium containing 5 μg/ml 6-thioguanine and no antioxidants. 0: medium without antioxidants, CAT: catalase, GSH: glutathione, SOD: superoxide dismutase. Error bars = standard error from 3–10 independent experiments each with 3–4 parallel dishes. *: significantly different from unirradiated cells (Mann-Whitney rank sum test, p < 0.05). ◆: significantly different from cells not treated with antioxidants (Mann-Whitney rank sum test, p < 0.05). Mutant frequency = number of mutant colonies per million cells seeded.

**Table 1:** Clonogenic survival (%) of cells incubated with antioxidants after exposure to 321 kJ/m^2 ^UVA- or 8.1 kJ/m^2 ^UVB-radiation

Antioxidant	Cells seeded before radiation^a^		Cells seeded 13 days after radiation^a^	
	
	UVA	UVB	UVA	UVB
None^b^	36 ± 12	33 ± 10	85 ± 7	80 ± 5
Catalase	21 ± 6	30 ± 5	84 ± 5	127 ± 12^d^
GSH^c^	19 ± 8	41 ± 13	127 ± 15	163 ± 26^d^
SOD^c^	28 ± 6	29 ± 4	135 ± 12	105 ± 9

^a^Mean values ± standard error from 3–10 independent experiments.

^b^The cloning efficiency of unirradiated cells treated with antioxidants for 6 days after seeding were not significantly different from untreated cells.

^c^The cloning efficiency for unirradiated cells treated with GSH or SOD for 13 days before seeding was significantly lower than for unirradiated cells not treated with antioxidants (t-test, p < 0.05).

^d^Significantly higher survival than irradiated cells not treated with antioxidants (t-test, p < 0.05).

UV radiation decreased the clonogenic survival of the cells significantly (t-test, p < 0.01), but none of the antioxidants increased the survival of the irradiated V79 cells significantly (Table 1). There were also no significant differences between the sizes of the colonies of cells that had been treated with antioxidants or untreated (data not shown). Both irradiated and unirradiated cells divided more often than every 24 hours [6]. However, UV radiation resulted in significantly smaller colonies than the unirradiated cells when measuring survival shortly after radiation ("Cells seeded before radiation"-columns of Table 1), which may indicate that irradiated cells grew slower than the unirradiated for a while after radiation. The clonogenic survival of UVA- and UVB-treated cells was slightly decreased compared to control cells 13 days after radiation, but not significantly, suggesting only a limited amount of delayed cell death. There was apparently no effect of the HAT treatment on clonogenic survival since there was no significant difference between the unirradiated cells seeded before radiation and unirradiated HAT treated cells seeded 13 days after radiation. However, there were some long-term effects of the antioxidants. For UVB radiation, catalase and GSH significantly increased survival. For unirradiated cells GSH and SOD significantly decreased the cloning efficiency, which suggest that long-term incubation with GSH and SOD may be toxic.

### Early UV-induced mutations

Early *hprt *mutations were measured after an expression period of 8 days in normal medium. Both 321 kJ/m^2 ^of UVA radiation and 8.1 kJ/m^2 ^of UVB radiation significantly increased the early mutant frequencies above control frequency (Figure 2). Treatment with antioxidants after irradiation had only minor effects on the early mutagenic effect of UVA and UVB radiation (Figure 2). However, GSH significantly reduced the early UVB-induced mutation frequency by 24 %, suggesting an oxidative component for early UVB-induced mutations. The antioxidants seemed to increase the background mutation frequency. However, the changes were not significant.

### Delayed UV-induced mutations

**Figure 3:**
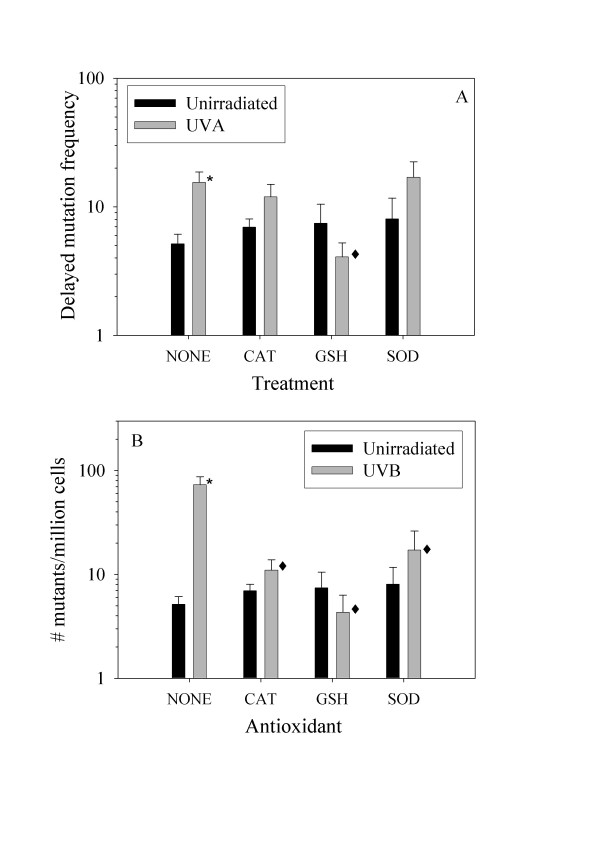
**Antioxidants inhibit UVA- and UVB-induced delayed mutagenesis**. Cells were exposed to 321 kJ/m^2 ^UVA- (A) or 8.1 kJ/m^2 ^UVB radiation (B). The cells were grown with or without antioxidants in HAT-medium for 8 days after ultraviolet irradiation to kill early HPRT mutants. Subsequently, the cells were subcultured and grown with antioxidants in ordinary medium for 5 days before seeding in medium containing 5 μg/ml 6-thioguanine and no antioxidants. 0: medium without antioxidants, CAT: catalase, GSH: glutathione, SOD: superoxide dismutase. Error bars = standard error from 3–10 independent experiments each with 3–4 parallel dishes. *: significantly different from unirradiated cells (Mann-Whitney rank sum test, p < 0.05). Mutant frequency = number of mutant colonies per million cells seeded.

Delayed *hprt *mutations were measured after 8 days in HAT medium to eliminate early mutations, followed by 5 days in normal medium. Both 321 kJ/m2 of UVA radiation and 8.1 kJ/m2 of UVB radiation significantly increased the delayed mutant frequencies above control frequency (Figure 3). Figure 3A shows that GSH significantly reduced the formation of delayed *hprt *mutations after UVA radiation by 74 % as compared with UVA radiation alone (p < 0.05). SOD and catalase had no significant effect on UVA induced delayed mutagenesis. Antioxidants had a greater effect on UVB-induced delayed mutations (Fig. 3B): catalase, GSH and SOD reduced the mutation frequency by 85 %, 94 % and 77 % as compared with UVB radiation alone, respectively (p < 0.05). Both catalase and GSH decompose H_2_O_2_. Thus, the results suggest that a persistent increase in H_2_O_2 _was involved in the formation of delayed mutations.

### Measurement of intracellular level of GSH

The fluorochrome monochlorobimane (MCB) was used to measure whether incubation with GSH could increase intracellular levels of GSH. Cells were treated with 5 mM GSH for 4 days, washed and stained with 50 μM MCB, which resulted in a significant increase in fluorescence. The fluorescence ratio between cells treated with GSH and untreated cells was 2.0 ± 0.4 (Mean ± SD).

## Discussion

We here report that both UVA and UVB radiation induced delayed *hprt *mutations in V79 Chinese hamster fibroblasts. Mutations induced several cell generations after UV radiation are an indication of genomic instability. The UVB-induced delayed *hprt *mutations were strongly inhibited by adding the antioxidants catalase, superoxide dismutase and GSH after irradiation, while only GSH had a significant effect on UVA-induced delayed mutations. The strong effects of these antioxidants indicate that oxidative stress has a major role in maintaining increased mutation rates long after exposure to UV radiation. The increased mutation rates of unstable cells may allow them to accumulate mutations that subsequently lead to cancer.

Recently, we showed that a much higher degree of bystander effect was involved in UVB-induced than in UVA-induced delayed mutagenesis [25]. UV-induced delayed mutations were measured with the same method as in the present paper, which allows for a high degree of cell-cell contact, and with a cloning method in which delayed mutations were measured in individual cell clones [6]. UVA- and UVB-induced delayed mutagenesis was 4 and 19-fold higher, respectively, when using the present method as compared with the cloning method. The difference in degree of bystander-induced delayed mutations between UVA and UVB radiation might be explained by the present results. If it is assumed that the factors mediating the bystander-induced delayed mutations are long-lived reactive oxygen species like H_2_O_2_, then antioxidants should inhibit the bystander effect. Only GSH inhibited UVA-induced delayed mutagenesis and GSH, SOD and catalase inhibited UVB-induced delayed mutagenesis (Fig. 3). Cells probably take up neither GSH, SOD nor catalase. However, breakdown of extracellular GSH can be initiated by the plasma membrane enzyme γ-glutamyltransferase (GGT), which removes the γ-glutamyl moiety, and the resulting products can be taken up by cells and stimulate intracellular GSH synthesis [31]. This hypothesis is supported by a two-fold increase in monochlorobimane-fluorescence from cells incubated with 5 mM GSH for 4 days as compared with control cells. Thus, to inhibit UVA-induced delayed mutagenesis the intracellular level of antioxidants has to be increased and the bystander effect may be assumed to be an effect that is transferred between generations of cells or via gap junctions. In fact, inhibition of gap junctional intercellular communication by dieldrin significantly decreased the induction of delayed mutations [25]. On the other hand, catalase and SOD, which are only effective outside the cells, inhibited UVB-induced delayed mutagenesis, but not as effective as GSH. Therefore, much of the UVB-induced bystander effect is suggested to be an effect that is transferred to neighbouring cells via the medium. This may be a mechanism of bystander induced delayed mutations only relevant for UVB radiation and that comes in addition to the bystander effect between related cells or via gap junctions. In conclusion, induction of delayed mutations may be divided into three categories: 1) Directly induced delayed mutations due to genomic instability caused by mutations in gatekeeper or caretaker genes [32]; 2) Induced by a bystander effect transmitted from generation to generation of related cells or via gap junctions; and 3) Induced by a bystander effect transmitted via the medium.

Catalase decomposes H_2_O_2 _to water and oxygen while GSH reduces a broader spectrum of oxidants, including H_2_O_2_. GSH decomposes H_2_O_2 _in a reaction catalysed by glutathione peroxidase and reacts with H_2_O_2 _downstream products. H_2_O_2 _is a weak oxidant and is poorly reactive [23]. Nevertheless, hydrogen peroxide and its more reactive downstream products, such as lipid peroxides and hydroxyl radical, may act as clastogenic factors [33] and thus produce delayed chromosome aberrations and mutations. Therefore, inhibition of UVB-induced delayed mutagenesis by catalase and GSH may indicate that H_2_O_2 _was produced in mutagenic concentrations in the period 8 to 13 days after irradiation. This conclusion is consistent with those of previous reports showing that hydrogen peroxide may induce genomic instability [34,35]. UVB-induced delayed mutagenesis was also inhibited by SOD. It has been shown that superoxide, which is converted to hydrogen peroxide by SOD, may act as a clastogenic factor and induce chromosome damage [37]. Thus, superoxide may also be involved in UVB-induced delayed mutagenesis. Furthermore, it has been demonstrated that medium harvested from V79 cells 22 hours after ultraviolet C exposure contains factors that can increase the mutation frequency of cells [36]. The identification of such clastogenic factors is clearly a key challenge for radiation research.

Using dihydrorhodamine 123 that become fluorescent when oxidized by ROS, including H_2_O_2_, genomically unstable cell clones exhibited significantly elevated levels of oxidative stress as compared to V79 control cells [38]. In accordance, ionizing radiation induced genomically unstable clones also had significantly increased levels of oxidative stress [39]. Interestingly, the ionizing radiation induced genomically unstable clones had elevated numbers of dysfunctional mitochondria with increased ROS production as compared to normal cells. Thus, it is tempting to speculate that UV-induced genomic instability also occurs via damage to mitochondria and a subsequent increase in oxidative stress. However, we have not been able to detect UV-induced persistent increase in oxidative stress in the whole population of V79 cells (J. Dahle, unpublished results), only in genomically unstable clones. Thus, it may be that only a small fraction of the irradiated cells gets an increase in the production of ROS, and that these cells are the same cells that have an unstable genome and get delayed mutations.

The antioxidants catalase, GSH and SOD decreased the UVB-induced delayed mutation frequency significantly but had almost no effect on the early UVB induced mutation frequency, except for a slight but significant effect of GSH on UVB-induced early mutations. The reason for this discrepancy may be differences in the types of early and delayed UV-induced DNA damage. Pyrimidine dimers, the main UVB-induced DNA lesion, are caused by direct absorption of UVB photons in DNA [10]. Thus, this type of damage cannot be inhibited by antioxidants added after irradiation, indicating that early and delayed UVB-induced DNA damage are different. This hypothesis is supported by the finding that UVB-induced delayed mutations, but not early mutations, are accounted for by large deletions [40], which are similar to the mutations produced by oxidative stress [41,42].

Pyrimidine dimer formation, oxidation of guanine and deletions are potential origins of early mutations after UVA-radiation [15,40,43,44]. The mechanism of oxidation of guanine probably involves singlet oxygen produced by UVA-activated photosensitizers [43]. Pyrimidine dimer formation by UVA radiation was reported after very high doses and the mechanism of induction may involve DNA damage via photosensitization [45]. The UVA absorbing chromophores have not been identified. Thus, because of the short life time of photosensitized reactions, the early DNA damage probably cannot be inhibited using antioxidants added up to 5 minutes after irradiation. In a multiplex PCR study it was shown that the majority of both early and delayed UVA-induced *hprt *mutants exhibited either total gene deletion or deletion of some of the exons of the gene [40]. Sequencing of delayed mutations might reveal further insight into the mechanisms of UV-induced genomic instability.

## Conclusion

In conclusion, UVB-induced delayed *hprt *mutations were strongly inhibited by antioxidants that eliminate H_2_O_2 _and superoxide anion, suggesting that a persistent increase of these reactive oxygen species or downstream products are involved in a bystander-induced formation of delayed mutations. This bystander effect may be mediated both via the medium and via gap junctional intercellular communication or transmitted from mother to daughter cells since CAT and SOD were not effective inside the cells, while GSH probably was effective both inside and outside the cells. The mechanism of UVA-induced delayed mutations may involve bystander-induced formation of delayed mutations mediated by gap junctional intercellular communication or transmitted from mother to daughter cells. UVA and UVB both induce delayed mutations – but are fundamentally different in how they interact with cells. Therefore, oxidative stress may also have a role in induction of delayed mutations induced by other types of stress than ultraviolet radiation.

## Abbreviations

GSH – glutathione

hprt – hypoxantine phosphoribosyl transferase

MCB – monochlorobimane

ROS – reactive oxygen species

SOD – superoxide dismutase

UVA – ultraviolet A (320 – 400 nm)

UVB – ultraviolet B (290 – 320 nm)

6TG – 6-thioguanine

## Authors' contributions

JD conceived the study, carried out the experimental work, performed the statistical analysis and wrote the manuscript. EK and TS participated in the design and coordination of the study and with writing of the manuscript. All authors read and approved the final manuscript.
